# Combination statin and chemotherapy inhibits proliferation and cytotoxicity of an aggressive natural killer cell leukemia

**DOI:** 10.1186/s40364-018-0140-0

**Published:** 2018-08-09

**Authors:** Austin B. Henslee, Timothy A. Steele

**Affiliations:** Department of Microbiology and Immunology, Des Moines University – College of Osteopathic Medicine, 3200 Grand Avenue, Des Moines, Iowa 50312 USA

**Keywords:** Aggressive natural killer cell leukemia, Statins, Chemotherapy, Cellular cytotoxicity, Cell cycle progression, ERK MAP kinase

## Abstract

**Background:**

Aggressive natural killer cell leukemia is a devastating disease, with an average patient survival time of less than 2 months following diagnosis. Due to P-glycoprotein-mediated resistance of the tumor cells most forms of chemotherapy are of limited efficacy, therefore new treatment strategies are needed. Statin drugs have recently been found to inhibit the growth of various tumor cell types.

**Methods:**

We investigated the effects of statin drug-mediated mevalonate pathway inhibition on cell proliferation, tumor-induced cytotoxicity, cell cycle progression and ERK MAP kinase signal transduction pathway activation. Flow cytometry was used to perform the cytotoxicity and cell cycle analyses and Western blotting was used to investigate ERK MAP kinase activation. Statistical significance was assessed by Student’s t-test.

**Results:**

Fluvastatin and atorvastatin were found to inhibit cell growth and tumor-induced cytotoxicity. These effects were reversed by the addition of mevalonate, signifying that the impact of the drugs were on the mevalonate pathway. Both drugs affected cell cycle progression by causing a significant increase in the percentage of cells in the G0/G1 phase and a reduction in the S phase and the G2/M phases of the cell cycle. Low concentrations of statin drugs were able to abrogate ERK MAP kinase pathway activation, which is typically constitutively activated in aggressive natural killer cell leukemias and important in tumor-mediated cytotoxicity. Addition of statins to chemotherapy caused enhanced inhibition of cell growth and cytotoxicity, compared to either agent alone; a combination therapy that could conceivably benefit some patients.

**Conclusions:**

These investigations suggest that inhibiting the mevalonate pathway might provide a more effective therapy against this deadly disease when combined with chemotherapy. Given that millions of people are currently taking statin drugs to lower cholesterol levels, the risk profile for statin drugs and their side effects are well-known. Our studies suggest that it may be beneficial to explore statin-chemotherapy combination in the treatment of aggressive natural killer cell leukemias.

## Background

As part of the innate immune response, natural killer (NK) cells are large granular lymphocytes that compose the first line of defense against virus infections [1] and are known to kill certain tumor cell types [2]. Therefore it is not surprising that NK cells may play a role in killing certain types of human tumors that have viral origins, such as those caused by Epstein-Barr virus, hepatitis B virus, hepatitis C virus and human papilloma virus [3]. NK cell-based antitumor therapies, using autologous or allogeneic NK cells, are being investigated as potential approaches to controlling, or potentially eradicating, patient tumor [4]. Newer discoveries about the characteristics and functions of NK cells include the immunoregulatory role of NK cell subsets [5] and how NK cells can develop a form of immunologic memory [6].

As is true of many human cells types, NK cell-derived leukemias can develop, albeit rarely compared to other forms of leukemia [7]. There are several forms of NK cell leukemia that are recognized by the World Health Organization as part of a larger group called large granular lymphocytic leukemias, including chronic NK cell lymphocytosis (provisionally recognized), aggressive NK cell leukemia (ANKL) and extranodal NK/T cell lymphoma, nasal-type and extranasal [8]. Therapy of ANKL patients with standard chemotherapy is consistently poor with one study demonstrating an average survival time of only 58 days following standard chemotherapy [9]. It was felt that the expression of the multidrug resistant efflux pump P-glycoprotein by ANKL cells contributed significantly to the resistance of ANKL cells to chemotherapeutic agents [10, 11]. Hematopoietic stem cell transplantation is an option for some ANKL patients, but only if tumor remission can be achieved with chemotherapy. Given the poor results with standard chemotherapy, ANKL patients need a more effective therapeutic approach.

**Fig. 1:**
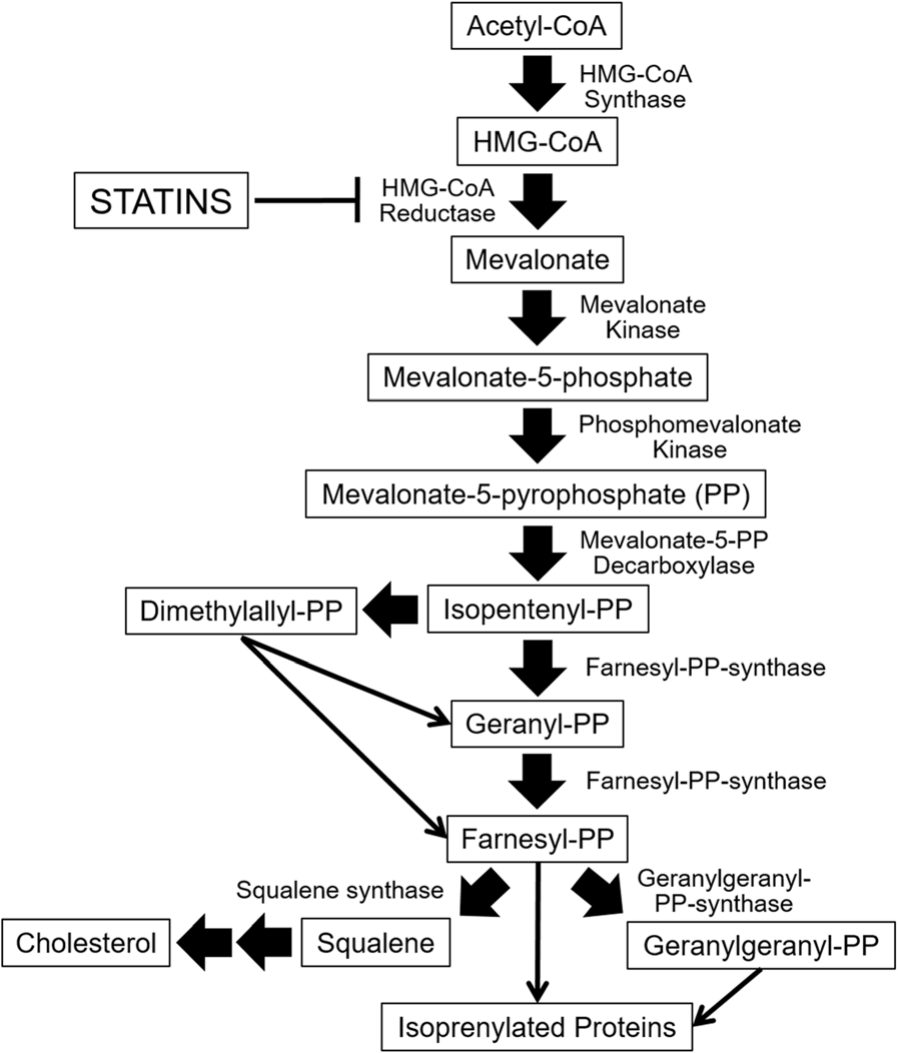
Mevalonate pathway. The diagram illustrates the mevalonate pathway that leads to the production of cholesterol and the farnesylation and geranylgeranylation of cellular components critical for the functioning of the cell

One promising experimental pre-clinical approach to cancer therapy has been to incorporate the use of statin drugs. Statins are commonly used for lowering cholesterol levels [12, 13]. This drug class inhibits HMG-CoA reductase in the mevalonate pathway (Fig. 1), thus blocking the synthesis of mevalonate and, ultimately, the production of cholesterol [14]. Beyond simply lowering cholesterol, some statins have shown antitumor activity with various forms of cancer, particularly gastrointestinal cancers [15–18]. In terms of leukemias, some statin compounds have shown pre-clinical activity against acute lymphoblastic leukemia [19] and chronic lymphocytic leukemia [20]. Our laboratory has shown that proliferation and cytotoxicity of the ANKL cell line YT-INDY could be inhibited by atorvastatin, fluvastatin or mevastatin and that the inhibition can be reversed by the addition of mevalonate or geranylgeranyl pyrophosphate [21].

The YT-INDY cell line, as a model for NK cell leukemias, was used in all of our experimental protocols. YT-INDY was cloned from the YT cell line and is independent on interleukin-2 or other additional cytokines. YT cells were obtained from a 15-year old acute lymphoblastic leukemia patient that ultimately died from his cancer [22]. Given that large granular lymphocyte leukemias can be derived from T lymphocytes, it is important to note that the YT cell line does not express typical T lymphocyte cell surface glycoproteins such as CD3, CD4 or CD8, but does express several surface markers associated with NK cells. Importantly, the T cell antigen receptor genes are in the germline configuration, signifying these cells are not from the T lymphocyte lineage [23].

In the current investigations, the YT-INDY cell line was used to determine whether statins could inhibit cell proliferation, cytotoxicity, cell cycle progression and ERK MAP kinase signal transduction pathway. Our studies showed that statins alone inhibited several important leukemic processes in YT-INDY and, in combination with certain chemotherapeutic agents, produced results significantly greater than either drug alone.

## Methods

### Chemicals

The chemotherapeutic agents used included doxorubicin (Fisher Scientific, Waltham, MA, USA), paclitaxel (LKT Laboratories Inc.. St. Paul, MN, USA) and topotecan (Toronto Research Chemicals, Toronto, Canada). The statin drugs used in the research include atorvastatin (Toronto Research Chemicals Inc., Toronto, Canada), fluvastatin (Selleck Chemicals, Houston, TX, USA), lovastatin (EMD Millipore, Billerica, MA, USA), mevastatin (EMD Millipore, Billerica, MA, USA), pravastatin (EMD Millipore, Billerica, MA, USA) and simvastatin (EMD Millipore, Billerica, MA, USA). Mevalonolactone (Sigma-Aldrich, St. Louis, MO, USA) was dissolved in RPMI-1640 medium (Fisher Scientific, Waltham, MA, USA) and titrated with NaOH to a pH of 7.8 to form mevalonate. Doxorubicin, fluvastatin and pravastatin were dissolved in distilled water. Paclitaxel, topotecan, and atorvastatin were dissolved in methanol. Lovastatin, mevastatin and simvastatin were dissolved in dimethyl sulfoxide (DMSO). The chemotherapeutic agents, statins, and mevalonate were aliquoted and stored at − 80 °C.

### Cells and cell culture

The YT-INDY and Phebo cell lines were cultured in RPMI-1640 media supplemented with 10% cosmic calf fortified bovine serum (Hyclone, GE Healthcare Bio-Sciences, Pittsburgh, PA, USA) and penicillin (100 units/mL)-streptomycin (0.1 mg/mL) (MP Biomedicals, Santa Ana, CA, USA) and grown at 37 °C and 5% CO_2_.

### Cell proliferation assay

200 μL of YT-INDY cells, at 100,000 cells/mL, were pipetted into each well of a 96 well plate and incubated at 37 °C and 5% CO_2_ with various concentrations of the statins fluvastatin or atorvastatin, or the chemotherapeutic drugs doxorubicin, paclitaxel or topotecan, or combinations of chemotherapy drugs and statins. The drug solvent vehicles used for the drug dilutions were included in the control wells. Cell proliferation of the YT-INDY cells was determined by trypan blue exclusion and hemocytometer counting at 72 h post-treatment. Each experiment was performed at least three times and the data was analyzed for statistical significance by Student’s t-test.

### Cellular cytotoxicity assay

YT-INDY were either treated with statins alone for 24 h or, for some experiments, chemotherapy alone, statins alone, or combination chemotherapy and statins for 72 h prior to testing in the cytotoxicity assay. The drug solvent vehicles were included in the control wells. The YT-INDY-sensitive tumor cell line Phebo, a B-cell leukemia line, was used as the target cell in all cytotoxicity assays. Approximately 6-8 × 10^6^ Phebo, suspended in 0.5 ml cosmic calf serum-supplemented RPMI-1640, were incubated for 90 min. at 37 °C in the presence of 200 uCi sodium chromate (^51^chromium; Perkin-Elmer, Inc., Waltham, MA, USA). Following incubation, the Phebo target cells were washed three times in Hank’s balance salt solution (Fisher Scientific, Waltham, MA, USA). Radiolabeled Phebo was added to wells of a 96-well plate, followed by treated or control YT-INDY cells to yield a 40:1 YT-INDY:Phebo ratio. The plates were centrifuged at 150 g for 2 minutes and then incubated for 4 h in a 37 °C, 5% CO_2_, humidified atmosphere. Following incubation, the plates were centrifuged at 150 g for 2 min. and the supernatant from each well harvested using a Supernatant Collection System (Molecular Devices, Sunnyvale, CA, USA). The harvesting filters containing the supernatant were placed within 12x75mm tubes, and the amount of chromium release was measured in a Packard Cobra Gamma Counter (Perkin-Elmer, Inc., Waltham, MA, USA).

The percent lysis of the Phebo target cells was computed as follows:

$$ \%\mathrm{lysis}=\kern0.5em \frac{\mathrm{test}\ \mathrm{cpm}-\mathrm{spontaneous}\ \mathrm{cpm}}{\mathrm{maximum}\ \mathrm{cpm}-\mathrm{spontaneous}\ \mathrm{cpm}}\kern0.5em \times \kern0.5em 100 $$

The maximum ^51^chromium contained in the target cells was determined by measuring the radioactivity in the radiolabeled Phebo. Measurement of radioactivity in the supernatant of wells containing only labeled target cells, following the 4 h assay, yielded the spontaneous release. Percent inhibition of cytotoxicity was derived by comparing the experimental value to the uninhibited control value. Each experiment was performed three times in quadruplicate to provide for statistical analysis by Student’s t-test.

### Cell cycle analysis

1 × 10^6^ YT-INDY, that had been incubated with or without fluvastatin or atorvastatin for 72 h, were washed three times in Hank’s balanced salt solution and labeled with propidium iodide (Sigma-Aldrich, St. Louis, MO, USA). The cells were analyzed by flow cytometry using a Becton-Dickison FACScan instrument (Franklin Lakes, NJ, USA). Quantitation of the percentage of cells in the G0-G1, S and G2-M phases of the cell cycle was accomplished using ModFit™ software (Topsham, ME, USA).

### Western blot analysis of ERK activation

Western blotting for total and phospho-ERK was conducted using statin-treated or control YT-INDY cells following a 72 h incubation at 37 °C and 5% CO_2_. Drug solvent vehicles were included in the control treatments. Following incubation, 1 × 10^6^ YT-INDY cells were placed in 12x75mm tubes. The cells were then centrifuged at 150 g for 7 min. The supernatant was removed from the cell pellets and 100 μL of lysis buffer (containing phosphatase- and protease-inhibitors) was added. Cells were transferred into microcentrifuge tubes and frozen at − 80 °C for 5 min. Then the cells were thawed on ice to undergo cell lysis. Finally, the tubes were microcentrifuged in order to pellet cell debris. The protein supernatant was transferred to a fresh microcentrifuge tube and the protein concentration was determined. SDS-PAGE was performed using a 12% polyacrylamide gel using a mini-gel electrophoresis system (Bio-Rad Laboratories, Hercules, CA, USA). Samples were heated at 96 °C for 5 min, cooled on ice, and then pulse-spun by centrifugation. Next, samples were loaded onto the gel and run at 200 V for 60 min. Following electrophoresis, the proteins were transferred to an Immobilon-P PVDF membrane (EMD Millipore, Billerica, MA, USA). The transfer was performed at 0.35 mA for 60 min. Following transfer, the membrane was briefly washed in tris buffered saline (TBS) to remove the transfer blotting buffer and then placed into blocking solution of 5% bovine serum albumin containing 1% Tween for 4 h. The membranes then underwent 3 washes with TBS-T (0.5% Tween) for 5 min before incubation with primary antibody (Santa Cruz Biotechnology). A 1:5700 and 1:250 dilution was used for the ERK and phospho-ERK primary antibodies, respectively. Primary antibody incubations were performed overnight on a rotating platform in a 4 °C cold room. After washing with TBS-T the following day, secondary antibodies (Santa Cruz Biotechnology, Dallas, TX, USA) were added. Goat anti-rabbit IgG-HRP was added to the ERK membrane at a 1:5000 dilution in 10 mL of 5% bovine serum albumin, and goat anti-mouse IgG-HRP was added to the p-ERK membrane at a 1:2000 dilution in 10 mL of 5% bovine serum albumin. After 1 h, washes with TBS-T (0.5% Tween), TBS, and distilled water were performed. Finally, the PVDF membrane bands were visualized by chemiluminescence (Pierce SuperSignal kit, Fisher Scientific, Waltham, MA, USA), and autoradiography.

### Statistics

Student’s t-test was used to determine statistical significance using InStat statistical software (GraphPad Software, Inc., La Jolla, CA, USA).

## Results

### Fluvastatin and atorvastatin inhibit YT-INDY proliferation and cytotoxicity

**Fig. 2:**
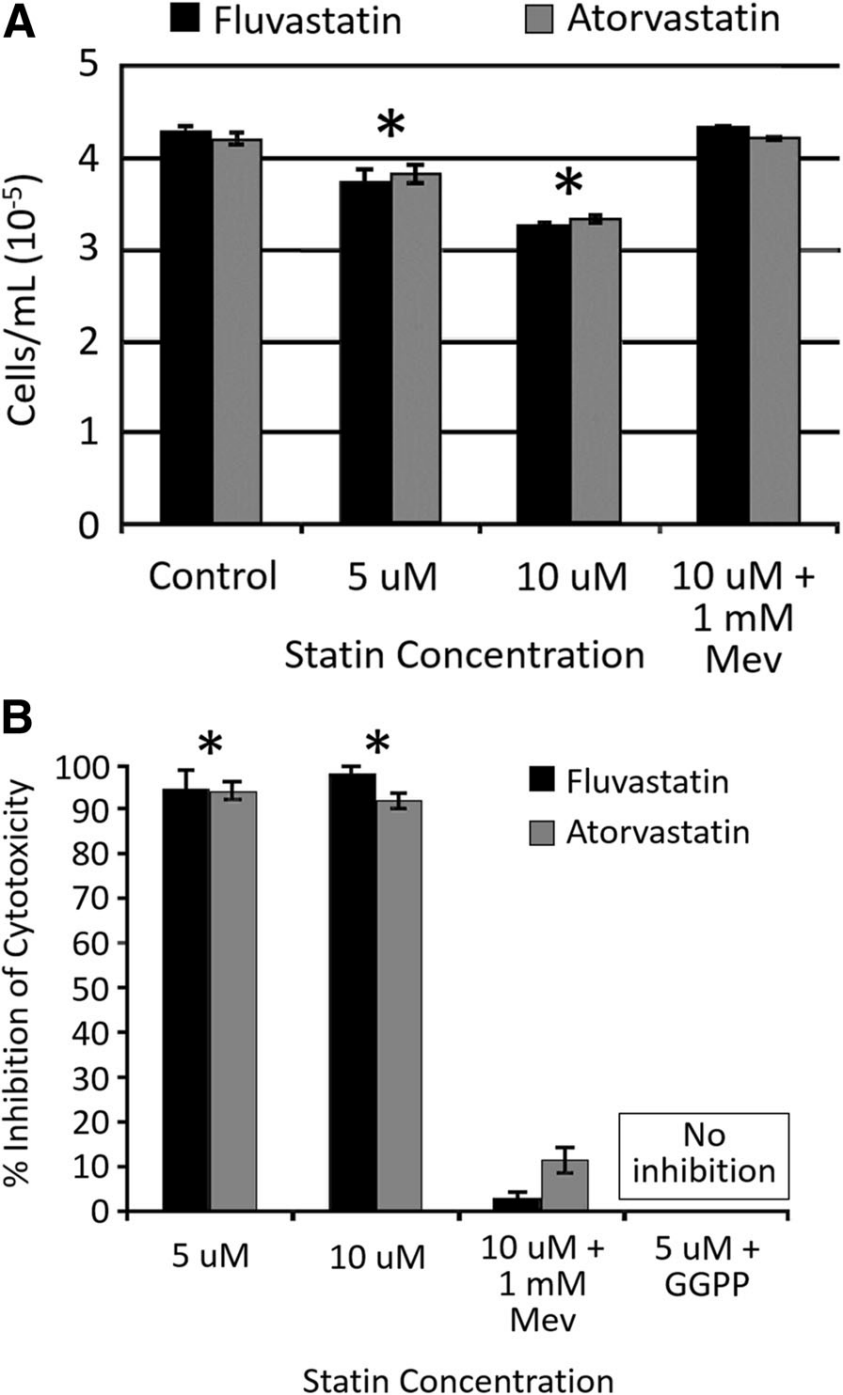
Fluvastatin and atorvastatin inhibit YT-INDY proliferation and cytotoxicity. Control cells were treated with the solvents in which the drugs were dissolved. All compounds were added to the cells at the start of the experiment. Each experiment was performed at least four times and the data was analyzed for statistical significance by Student’s t-test. Statistical significance at *p* < 0.05 is denoted by an asterisk. **a** YT-INDY cells were incubated for 72 h in the presence of fluvastatin or atorvastatin, in the presence or absence of mevalonate. These results show that fluvastatin and atorvastatin can inhibit YT-INDY cell growth and that mevalonate can reverse the effects of the statins, demonstrating that the major effect is taking place within the mevalonate pathway. **b** YT-INDY cells were incubated for 24 h with fluvastatin or atorvastatin, in the presence or absence of mevalonate or geranylgeranyl pyrophosphate, and tested in a cellular cytotoxicity assay. Control levels of YT-INDY cytotoxicity against the target cell line averaged 61.6% ± 3.32 (standard error of the mean) (*n* = 16 experiments). Experimental results were compared to control results to derive the percent inhibition of cytotoxicity. Fluvastatin and atorvastatin potently inhibited the cellular cytotoxicity of YT-INDY cells, whereas mevalonate and geranylgeranyl pyrophosphate could nearly completely restore cytotoxic activity

In order to confirm that the YT-INDY cell line was susceptible to the effects of statin drugs, we performed a proliferation assay using fluvastatin and atorvastatin concentrations of 5 μM and 10 μM for each compound. Following a 72 h incubation, it was observed that each concentration of the drugs produced a statistically significant reduction in proliferation of YT-INDY (Fig. 2a). To verify that the effects of fluvastatin and atorvastatin were influencing the mevalonate pathway directly, rather than indirectly affecting other metabolic or signal transduction pathways, we were able to restore proliferation to control levels using 1 mM mevalonate at the highest used statin drug concentration (10 μM).

Next, we confirmed that 5 μM and 10 μM fluvastatin and atorvastatin could nearly abolish the cytotoxicity of YT-INDY cells against a susceptible target cell line (Fig. 2b). Addition of 1 mM mevalonate or 10 μM geranylgeranyl pyrophosphate could restore nearly all of the cytotoxicity of YT-INDY cells, demonstrating that inhibitory effect of the drugs was taking place at the level of the mevalonate pathway. These results demonstrate that the YT-INDY cell line is sensitive to the disruption of the mevalonate pathway involving proliferation and cytotoxicity.

### Fluvastatin and atorvastatin inhibit the cell cycle of YT-INDY cells and reduce cell size

**Fig. 3:**
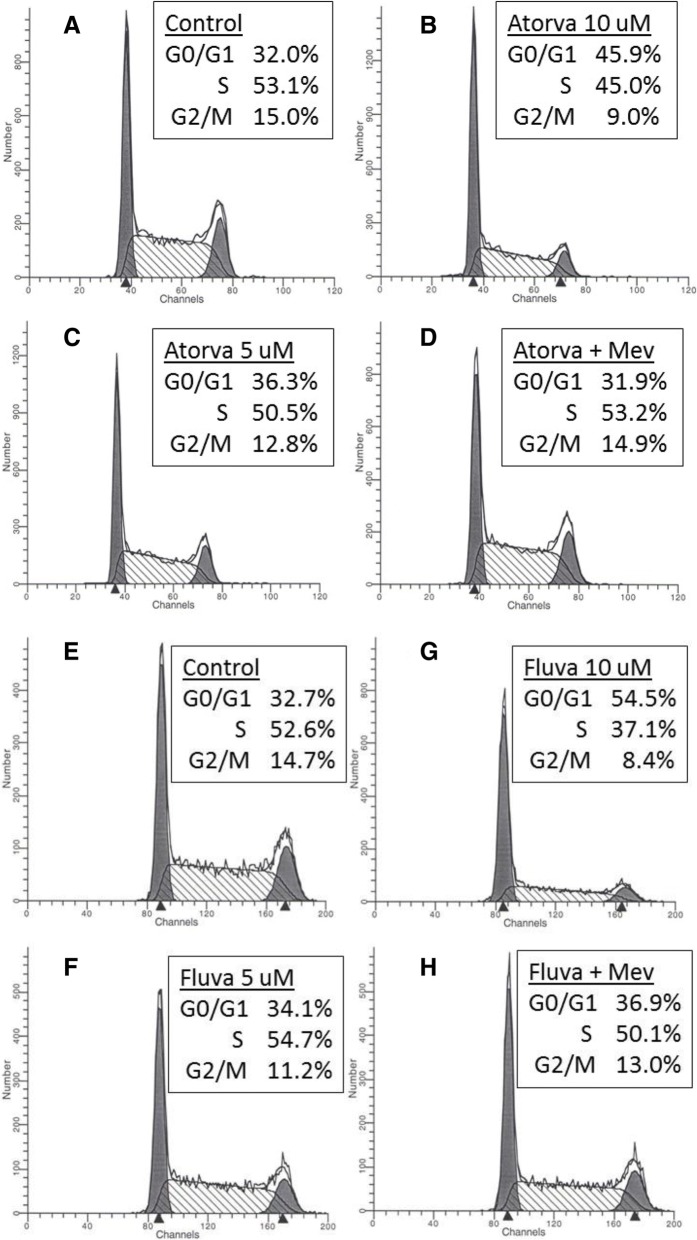
Statins exert an inhibitory effect on the cell cycle. YT-INDY cells were treated with either fluvastatin, atorvastatin or vehicle for 72 h, then labeled with propidium iodide and analyzed by flow cytometry. Data reflect a representative experiment. Values represent the percentage of cells in each phase of the cell cycle. **a**, **e** Control YT-INDY cells were treated with vehicle only. **b**, **c**, **g**, **f** YT-INDY cells were treated with either 10 uM or 5 uM atorvastation or fluvastatin alone. **d**, **h** YT-INDY cells were treated with either 10 uM of atorvastatin or fluvastatin and 1 mM of mevalonate

To more fully explore the inhibitory effect of fluvastatin and atorvastatin on proliferation, we investigated the effects of these compounds on the progression of the YT-INDY cell cycle using flow cytometry. At a drug concentration of 10 μM, both fluvastatin and atorvastatin produced a statistically significant (*p* < 0.05) increase in the percentage of cells in the G0/G1 phase and a reduction in the S phase and the G2/M phases of the cell cycle (Fig. 3). At a drug concentration of 5 μM, there were slight, although not statistically significant, effects on the various phases of the cell cycle by the two drugs. Reversal of the statin effect was observed when 1 mM of mevalonate was present. Overall, the results suggested that an inhibitory effect on the cell cycle may play a role in the reduction of YT-INDY proliferation.

Interestingly, a 72-h incubation of YT-INDY cells with fluvastatin and atorvastatin resulted in a significant and concentration-related decrease in cell size (Table 1) when forward scatter was analyzed by flow cytometry. The relevance of this finding will require further investigation.

**Table 1:** Statins cause a reduction in cell size^a^

Statin	Treatment	FSC ± SEM
Fluvastatin	Control	175 ± 7.4^b^
	5 uM	**156 ± 8.4**
	10 uM	**136 ± 7.1**
Atorvastatin	Control	178 ± 8.8
	5 uM	**155 ± 2.1**
	10 uM	**153 ± 2.8**

^a^YT-INDY cells were treated with either fluvastatin or atorvastatin for 72 h then analyzed by flow cytometry for forward scatter. Control cells were treated with vehicle only

^b^Data represent the mean forward scatter values ± standard error. Bolded values represent statistical significance at p < 0.05 compared to controls

### Statins inhibit the ERK MAP kinase pathway in YT-INDY

**Fig. 4:**
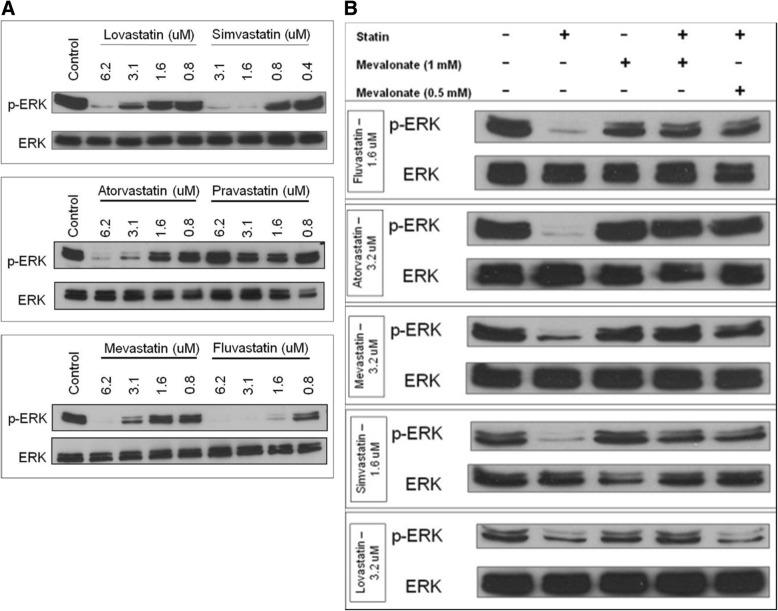
Statins inhibit the ERK MAP kinase pathway in YT-INDY cells. YT-INDY cells were incubated with various concentration of statins, followed by Western blotting for ERK and phospho-ERK. Control cells were treated with the solvents in which the drugs were dissolved. All compounds were added to the cells at the start of the experiment. Each experiment was performed at least four times. **a** The results demonstrate that lovastatin, simvastatin, atorvastatin, mevastatin and fluvastatin could inhibit ERK MAP kinase pathway activation at relatively low drug concentrations, whereas no inhibition of the pathway was observed with pravastatin. This was likely due to the hydrophilic nature of the drug preventing it from crossing the YT-INDY cell membrane. **b** Using the five statins that inhibited the ERK MAP kinase pathway, the ability of mevalonate (1 mM or 0.5 mM) to restore the pathway was determined. Our results showed that 1 mM mevalonate was capable of restoring all statin-mediated inhibition of the ERK MAP pathway and 0.5 mM was sufficient to restore activity for all statins except lovastatin

Given the importance of the ERK MAP kinase pathway in cell proliferation, we explored the effects of the statins on YT-INDY ERK MAP kinase pathway activation. Figure. 4a shows the effect of very low concentrations of statin drugs on the inhibition of the ERK MAP kinase pathway in YT-INDY cells. Lovastatin, simvastatin, atorvastatin, mevastatin and fluvastatin could inhibit ERK MAP kinase pathway activation at relatively low drug concentrations, whereas no inhibition of the pathway was observed with pravastatin. This was likely due to the hydrophilic nature of the drug preventing it from crossing the YT-INDY cell membrane. Mevalonate at a concentration of 1 mM or 0.5 mM was sufficient to abrogate the inhibitory effect of nearly all statins used (Fig. 4b). These results show that at least one of the downstream effects of mevalonate pathway inhibition involves the ERK MAP kinase pathway.

### Combination of statins and chemotherapy augment the inhibition of proliferation and cytotoxicity

**Fig. 5:**
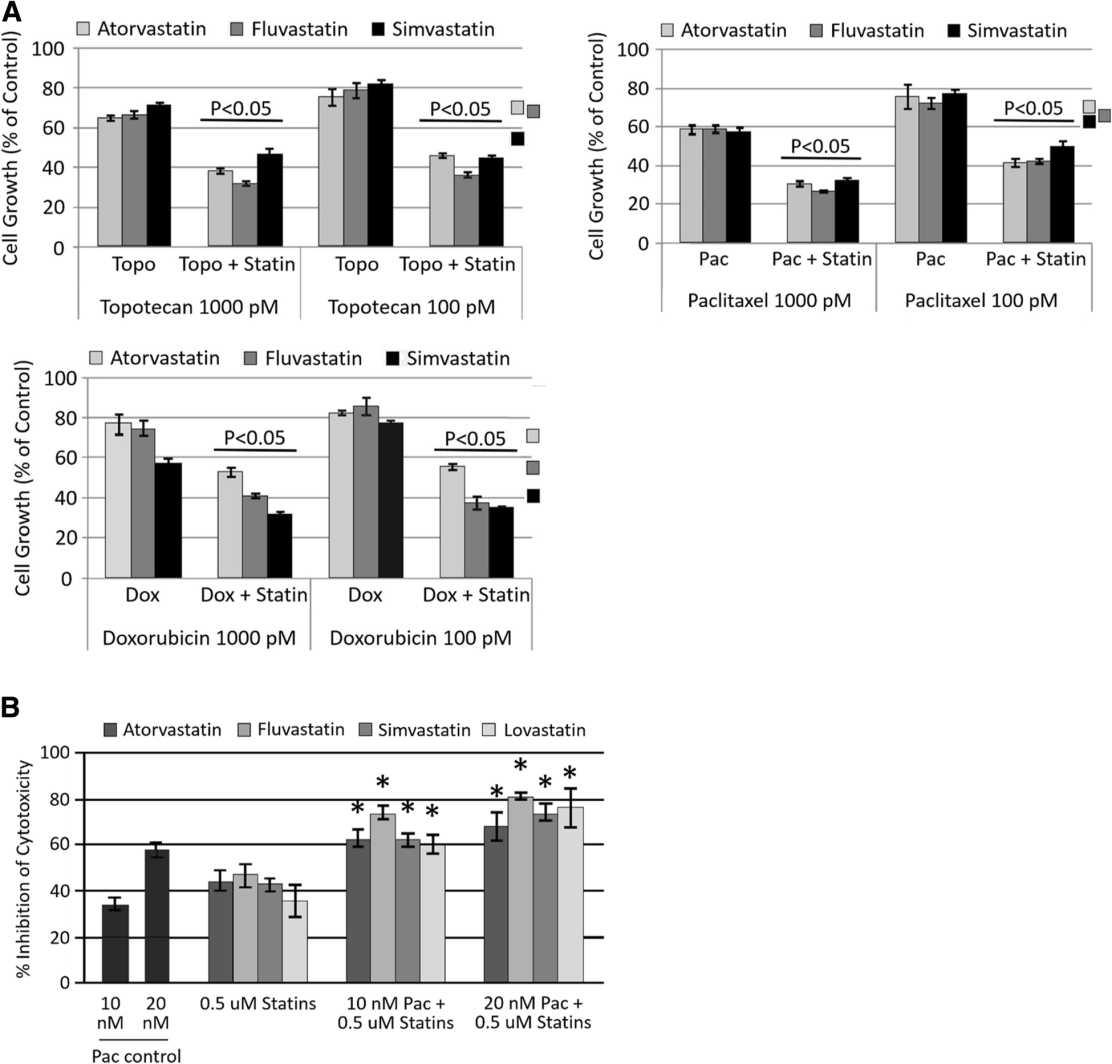
Combination of statins and chemotherapy augment the inhibition of proliferation and cytotoxicity. YT-INDY cells were incubated with topotecan, paclitaxel or doxorubicin in the presence or absence of statins for 72 h. Cell growth and cellular cytotoxicity was measured and expressed as a percent of cell control, which was treated with the solvent in which the drug was dissolved. **a** Addition of atorvastatin, fluvastatin or simvastatin to the chemotherapy drugs enhanced the inhibition of cell growth compared to either drug alone at a statistical significance level of *p* < 0.05 when compared to the single drug controls. **b** Addition of statins to paclitaxel-treated YT-INDY demonstrated an enhance inhibition of cellular cytotoxicity, which was statistically significant at the p < 0.05 level for all statin/paclitaxel combinations. A similar effect was not observed with topotecan or doxorubicin. Control levels of YT-INDY cytotoxicity against the target cell line averaged 61.6% ± 3.32 (standard error of the mean) (*n* = 16 experiments)

Since there is a great need for better therapy for ANKL patients due to the intrinsic resistance of the ANKL cells to chemotherapeutic agents, we tested whether statins would provide an enhanced or synergistic effect when used in combination with chemotherapy drugs. As show in Fig. 5a, the use of various statins in combination with either doxorubicin, paclitaxel or topotecan provided a greater inhibitory effect on the proliferation of YT-INDY cells than either compound alone.

A similar effect was observed on YT-INDY cytotoxicity, but only with paclitaxel (Fig. 5b) (doxorubicin and topotecan data not shown). Combined, these results suggest that statin and chemotherapy combinations might be used effectively in patients to increase the effectiveness of the chemotherapeutic agents. Given that some chemotherapy agents do not produce an enhanced effect when combined with statins, this approach might be limited against ANKL cytotoxicity. However, significant inhibition of cytotoxicity was achieved in our experiments with statins alone and in low concentration (Fig. 5b), so an enhanced effect with chemotherapy might not be essential to abrogate ANKL cytotoxicity.

## Discussion

These investigations showed that statins alone could inhibit cell proliferation, cytotoxicity, cell cycle progression and ERK MAP kinase activation. In addition, statins, in combination with certain chemotherapeutic agents, produced inhibitory effects on proliferation and cytotoxicity that were significantly greater than either drug alone. Given the typical poor outcome of chemotherapy against ANKL [9], it is imperative that more efficacious treatment strategies be explored. Our pre-clinical results suggest that statins, used with some types of chemotherapy, should be investigated potentially to provide a more effective therapy against ANKL.

After confirming that YT-INDY proliferation and cytotoxicity was significantly inhibited by fluvastatin and atorvastatin, we showed that treatment with either statin caused the accumulation of cells in the G0/G1 phase and a consequent reduction in cells that were in the S or G2/M phase of the cell cycle. We interpreted these results to mean that both statins likely blocked the G1/S transition. Reversal of this effect by the addition of mevalonate confirmed that the statins were working through the mevalonate pathway by inhibiting HMG-CoA reductase.

In Fig. 2b we showed that the inhibition of YT-INDY cytotoxicity was dependent on the inhibition of geranylgeranylation. Tanaka et al. demonstrated that inhibition of normal NK cell cytotoxicity was inhibited by simvastatin and fluvastatin, likely by compromising the exocytosis pathway and suppressing the release of granzyme B, a component important in the destruction of target cells [24]. Poggi et al. showed that fluvastatin, among other things, inhibited the activation of RhoA which is required for actin redistribution and the release of perforin and granzyme B by degranulation [25]. Rho A synthesis requires geranylgeranylation and may be a component in YT-INDY cells that is inhibited by fluvastatin and atorvastatin and which can be reversed by the addition of geranylgeranyl pyrophosphate (Fig. 2b).

One observed morphological change in statin-treated YT-INDY cells was a reduction in cell size, as measured by flow cytometry and analysis of forward scatter values (Table 1). The effect was more pronounced in fluvastatin-treated cells. The significance of this observation is unclear. The reduction in cell size could potentially be due to increased autophagy, but inhibition of the mevalonate pathway has been shown to increase cell size by blocking the maturation of autophagosomes by reduced geranylgeranylation of RAB11, a small GTPase that is required for maturation of autophagosomes [26]. Clinically, patients with large granular lymphocyte leukemias tended to have significantly smaller cells if their cells demonstrated STAT3 mutations in the SH2 domain compared to patients whose cells did not have STAT3 mutations [27].

Though not likely inhibiting the pathway directly, five statins had a profound inhibitory influence on the activation of the ERK MAP kinase pathway, which was reversed by the addition of mevalonate. The YT-INDY cell line constitutively expresses high levels of phosphorylated ERK as demonstrated in the controls show in Fig. 4a. Statins have been found to decrease activation of the ERK MAP kinase pathway in lymphoma [28] and myeloma cells [29] likely as a result of a decrease in the amount of Ras located at the plasma membrane. Interestingly, Liang, et al. demonstrated that in YT cells (parental line of the YT-INDY clone) cytotoxicity, but not cell proliferation, was dependent on ERK MAP kinase pathway activation [30]. This was likely due to the fact that proliferation utilized the NF-κB pathway in the YT cell line rather than the ERK MAP kinase pathway as was used for cell-mediated cytotoxicity. Proliferation was inhibited in the presence of NF-κB signaling pathway blockade, but not with an ERK MAP kinase pathway inhibitor. Therefore, this indicates that statin effects on proliferation likely occur independently of inhibition of the ERK MAP kinase pathway. In support of blocking the mevalonate pathway to decrease the amount of ANKL-related cytotoxic pathology, Epling-Burnett, et al. utilized the farnesyltransferase inhibitor tipifarnib to treat a patient with natural killer large granular lymphocyte leukemia [31]. This treatment resulted in improved patient pulmonary hypertension through blocking the ERK MAP kinase pathway signaling pathway for leukemic cell cytotoxicity of pulmonary endothelial cells.

Another potential avenue for therapy of ANKL are the MAPK/ERK kinases (MEK) inhibitors. In cancer therapy MEK inhibitors are increasingly being tested to induce tumor apoptosis, but acquired resistance to these inhibitors must be overcome. It has been shown that inhibiting the mevalonate pathway using fluvastatin or simvastatin can enhance tumor sensitivity to MEK inhibitors resulting in tumor apoptosis [32].

Although statins alone can have anti-tumor effects, ANKL cells are typically resistant to many forms of chemotherapy. Therefore, it was of interest to determine whether statins in combination with chemotherapy would provide a greater benefit compared to chemotherapy alone. Our investigations showed that doxorubicin, paclitaxel or topotecan, in combination with atorvastatin, fluvastatin or simvastatin produced greater inhibition of YT-INDY proliferation than either agent alone. Pre-clinical tumor models have shown that combination statin and chemotherapy can produce synergistic or enhanced effects compared to single agents only. Beneficial effects of this type of combination therapy on tumor cells have been shown against the hematopoietic tumors including chronic lymphocytic leukemia [33], acute leukemia [34, 35] and relapsed acute myelogenous leukemia [36].

ANKL cells tend to be resistant against most forms of chemotherapy due to expression of the P-glycoprotein multidrug resistance efflux pump [10, 11]. Doxorubicin is a substrate for P-glycoprotein. Interestingly, statins can inhibit P-glycoprotein via a nitric oxide mechanism [37, 38], possibly accounting for the enhanced effect of statin and doxorubicin combination.

In contrast to the effect of combination therapy on cell growth, only paclitaxel and statin combinations significantly inhibited YT-INDY cytotoxicity against a target cell. This may not be clinically significant given that statins alone exert significant inhibition of YT-INDY cytotoxicity (Fig. 2b), so relying on a combination effect with chemotherapy may not be necessary.

Recently, the role of the JAK/STAT pathway has emerged as being a potentially important part of the molecular pathogenesis of large granular lymphocytic leukemias. STAT3 gain-of-function mutations are found in a significant proportion of natural killer cell leukemias and may serve in the future as a therapeutic target for these leukemias [39–42]. Mutated STAT5b also has been observed in some large granular lymphocytic leukemias, though in a substantially smaller number of patient tumors [43]. STAT3 and STAT5b gene mutations also have been demonstrated in ANKL cells [44, 45]. Interestingly, statins have been shown to inhibit JAK/STAT activation in various tumor models. Simvastatin was shown to suppress phosphorylation of JAK2 and STAT3 in two human renal cell carcinoma cell lines in a nude mouse model [46] and growth hormone-stimulated JAK2 and STAT5 activation in osteosarcoma cells [47]. Atorvastatin-treated squamous cell carcinoma cell lines, derived from head and neck cancers involving the floor of the mouth and tongue, demonstrated a significant inhibition of phosphorylated STAT3 in connection with reduced metastatic potential [48]. It is unclear whether the statin-mediated inhibition of growth and cytotoxicity in our ANKL cell line is as a result of disturbed JAK/STAT signaling, but our laboratory has plans to investigate the potential link.

## Conclusions

The conclusions to these investigations are two-fold. The first is that statin drugs alone exert a significant inhibitory effect on the growth and cytotoxicity of our ANKL cell line, suggesting that these drugs might be useful in patients diagnosed with ANKL. Secondly, statins used in combination with various chemotherapy agents demonstrated an enhanced inhibition of both growth and cytotoxicity compared to either agent alone. Since ANKL cells typically are highly resistant to many forms of chemotherapy, due to the expression of P-glycoprotein, addition of statins to certain chemotherapy regimens might make them more efficacious against this deadly leukemia.

## Abbreviations

ANKL: Aggressive natural killer cell leukemia
DMSO: Dimethyl sulfoxide
NK cells: Natural killer cells
